# A short segment in the HIV-1 gp120 V1/V2 region is a major determinant of neutralization resistance to PG9-like antibodies

**DOI:** 10.1186/1742-4690-9-S2-O29

**Published:** 2012-09-13

**Authors:** NA Doria-Rose, I Georgiev, RP Staupe, S O'Dell, G Chuang, J Gorman, JS McLellan, M Pancera, M Bonsignori, BF Haynes, DR Burton, WC Koff, PD Kwong, JR Mascola

**Affiliations:** 1 National Institutes of Health, Bethesda, MD, USA; 2 Duke University, Durham, NC, USA; 3 The Scripps Research Institute, La Jolla, CA, USA; 4 International AIDS Vaccine Initiative, New York, NY, USA

## Background

Antibody PG9 is a prototypical member of a class of V1/V2-directed antibodies that effectively neutralizes diverse strains of HIV-1. The crystal structure of PG9 bound to scaffolded V1/V2 has provided insight into its mode of recognition. We sought to gain a more complete understanding of the interaction of PG9 with the functional viral spike, and to extend our understanding to other antibodies of this class.

## Methods

We analyzed amino acid frequencies in the V2 region of PG9 sensitive and resistant strains to identify potentially important residues. We also used the crystal structure of PG9 with scaffolded V1/V2 to identify potential contact sites. Based on these analyses we designed mutations in PG9 resistant strains with the goal of “knocking in” sensitivity. Parent and mutant Envs were used to make pseudoviruses. The potency of PG9 as well as V1/V2 antibodies PG16, CH01, CH04, PGT141, and PGT145 against the pseudoviruses was assessed by TZM-bl neutralization assay.

## Results

For 20/20 of resistant strains, mutations in a short segment of V1/V2 resulted in gain of sensitivity to at least one antibody of the PG9 class, and 13/20 showed gain of sensitivity to 3 or more. Mutations in V2 strand C, particularly the addition or substitution of lysine at positions 168, 169, or 171, had the greatest effects.

## Conclusion

These results highlight the importance of strand C contacts for neutralization by V1/V2 antibodies, provide functional confirmation of the crystal structure, and suggest a general mechanism of resistance to V1/V2-directed broadly neutralizing antibodies.

